# Comparable to 17α- methyl testosterone, dietary supplements of *Tribulus terrestris* and *Mucuna pruriens* promote the development of mono-sex, all-male tilapia fry, growth, survival rate and sex-related genes (*Amh, Sox9, Foxl2, Dmrt1*)

**DOI:** 10.1186/s12917-024-04162-0

**Published:** 2024-07-19

**Authors:** Aya F. Matter, Walaa S. Raslan, Eman I. Soror, Eman K. Khalil, Amgad Kadah, Hadeer A. Youssef

**Affiliations:** 1 Department of Aquatic Animal Medicine, Faculty of Veterinary Medicine at Moshtohor, Benha University, Benha, Egypt; 2 Department of Physiology, Faculty of Veterinary Medicine at Moshtohor, Benha University, Benha, Egypt; 3 Department of Anatomy and Embryology, Faculty of Veterinary Medicine at Moshtohor, Benha University, Benha, Egypt

**Keywords:** Nile tilapia, Monosex, *Tribulus terrestris*, *Mucuna pruriens,*sex related genes, 17α-methyl testosterone

## Abstract

To evaluate *Tribulus terrestris* and *Mucuna pruriens* for inducing all-male tilapia, mixed-sex Nile tilapia, *Oreochromis niloticus,* (mean weight 0.025 ± 0.009 g; mean length 1.25 ± 0.012 cm)*,* were given a meal supplemented with either *T. terrestris* powder (commercial fish feed, 40% crude protein) (TT group), *M. pruriens* seed extract (MP group), MP + TT (mixed group), 17α-methyl testosterone (MT, control positive), or without supplements (control negative). The MP extracts significantly increased (*P* < 0.05) the final weight, weight gain, weight gain rate, and specific growth rate while feed conversion ratio was significantly decreased (*P* < 0.05). Plant extracts markedly improved (*P* < 0.05) the survival rate, proportion of males, and total testosterone compared to control and MT. Estrogen levels were lower in groups with plant extract than other groups. Fifteen days post-feeding, the *Amh* gene was expressed in the brain of *O. niloticus* fries with higher levels in MP, TT, and MT groups. Additionally, the expression of the *Sox9* and *Dmrt1* genes as a male related genes in fish fry gonads revealed significantly (*P* < 0.05) higher levels in groups fed on MP, TT, and MT compared to control after 30-day post-feeding, whereas; *Foxl2* gene expression as a female related gene was significantly (*P* < 0.05) lower in fish fed on MP, TT, and MT compared to other groups after 30 days post feeding. Histologically, MT, MP, TT, and the mixture all exhibited solely male reproductive traits without noticeable abnormalities. This study concluded that each of the TT or MP extracts can induce sex reversal in tilapia while having no negative health impact compared to MT as the growth and survival rate in the treated groups with TT and MP were higher than control and group treated with MT.

## Introduction

Nile tilapia is the most widely cultivated fish in Egypt and has a high level of marketability. After the carp fish, it is regarded as the second-most significant species of fish raised for human use worldwide [1]. When kept in higher stocking density, female Nile tilapia demonstrate limited somatic growth and high fecundity, whereas male tilapia grows more quickly and are commonly preferred in monosex aquaculture [2]. The synthetic steroid methyl testosterone (MT) is widely utilized due to the steady rise in demand for mono-sex tilapia, which could have a negative impact on the environment [3]. Consequently, in order to produce monosexual tilapia, efforts are being undertaken to use an alternative, secure natural plant or herbal extracts [4]. Additionally, sex-reversed tilapia exhibits greater growth performance than regular tilapia. According to research by [5], fish sex reversal may be induced using phytochemicals since they are reported to limit the estrogen production and activity in gonad germ cells by acting as nuclear estrogen receptor antagonists and aromatase inhibitors.

In aquaculture, using a variety of feed additives as probiotics and phytobiotics reduces the usage of antibiotics and other chemicals [6, 7]. Due to their largely innocuous natural components, phytobiotics (plant additives) have recently gained popularity as aquafeed additives in fish farms in an effort to increase the performance of fish, and feed conversion 30. A medicinal plant called *Tribulus terrestris* affects androgen metabolism to increase levels of testosterone or testosterone precursors, [8]. The Tropical legume *Mucuna pruriens* is a widely cultivated and naturally occurring plant which originated in tropical Asia and Africa, [9]. Japanese flounder grew faster and used their fatty acids more effectively thanks to the herbal combination of these plants, [10]. The earliest indications of gonadal sex differentiation can be seen under a light microscope where the ovarian chamber in the XX gonad or the efferent duct in the XY gonad forms in tilapia fry between the ages of 23 and 26 days, [11]. The Anti-müllerian Hormone (*Amh*) is a hormone that inhibits the growth of müllerian ducts in female embryos, which would otherwise give rise to the uterus, fallopian tubes, and upper vagina, [12]. During the differentiation of the testis in male embryos in mammals, birds, and reptiles, despite the absence of müllerian ducts in teleosts, *Amh* orthologues have been discovered in these creatures. Determine the molecular make-up of *Amh*, if it is expressed in the brain, and when it is expressed in relation to gonadal expression during the sex differentiation and brain development of tilapia [13, 14].

In this study, we investigated a female gene expression involved in ovarian development Forkhead box transcription factor L2 (*Foxl2*) and two genes known to be Sertoli cell factors in male gonads Doublesex and Mab-3 related transcription factor 1 (Dmrt1) and SRY-Box Transcription Factor 9 (*Sox9*). To our knowledge, this research is the first to observe how Nile tilapia fries had boosted growth, survival, and sex reversal when fed on *T. terrestris* and *M. pruriens* corporate diets.

## Materials and methods

### Fish

The freshly hatched mixed-sex Nile tilapia fries were supplied from the fish hatchery in the Kafr El-sheikh government. They were carried in oxygen-packed containers and had average weights (0.025 ± 0.009 g) and lengths (1.25 ± 0.012 cm), and transported to the laboratory of Aquatic Animal Medicine Department, Faculty of Veterinary Medicine, Moshtohor Benha University. Fish were given a commercial fish meal that contained 40% crude protein and was delivered orally to sexually undifferentiated fries. Fish were kept in continually aerated 10 L well-prepared glass aquariums (kept at pH of range 7.3–7.7) and dissolved oxygen concentration of 5.0 mg/L in heated static systems (T = 27 ± 2 ^₀^ C). The fish were kept in aquariums with daily water changes and identical photoperiods (14 L: 10 D). The fish were split into five experimental groups with 100 fries each in triplicate after just one day of acclimation (5 × 100 × 3 = 1500 fries).

### Plant extracts and experimental design

*T. terrestris* and *M. pruriens* extracts were purchased in powder form Bulk Supplements, USA. Diets with these extracts at concentrations of 0.0 (control negative group), 2.0 g T*. terrestris*/kg feed (TT group), 2.0 g M*. pruriens*/kg feed (MP group), 1.0 g TT + 1.0 g MP/ kg feed (mixed group, Mix), and a diet containing 60 mg MT/kg feed (control positive group, MT group) [15]. The distribution of fish in 10-L glass aquariums was random (100 fish per aquarium; two aquariums were assigned for each treatment category, including controls positive and negative groups). Plant extracts in the necessary concentrations were dissolved in dimethyl sulfoxide (DMSO), which was then added to a finely powdered (500–1000 lm) artificial diet with 40% crude protein (6th October city, Egypt), [16]. Only DMSO was used as the control feed, and the artificial diet was coarsely powdered. The fish food was made by carefully mixing all the ingredients for 15 min before adding oil and water to make a wet, doughy mass. The dough mass was then pelleted without the use of steam, resulting in sinking pellets 2 mm in diameter. Finally, the pellets were dried at room temperature using the procedures of and stored in clean, sterile plastic bags at -20 °C until use. The pelleted meal was crushed up before being fed to the fish. For 30 days, fish were given their corresponding meals twice daily at a rate of 20% of body weight according to fry body weight. The average length, weight, male ratio, and survival rate were determined at the end of the experiment for each treatment.

### Determination of growth parameters and survival rate

Every 15 days post feeding, 20 fries from each aquarium were immersed in the anesthetic solution bath containing 125 mg/L tricaine methanesulfonate (MS222) (Ambion, USA) [17] which applied directly to the water (Syndel, British Columbia) for 3 min. The fries were weighed, and their lengths were measured using a digital electronic balance and ruler to assess several growth performance factors. The following metrics were evaluated: weight gain (WG), specific growth rate (SGR), weight gain rate (WGR), feed conversion ratio (FCR), and survival rate according to [18, 19].

$$\mathrm{Feed}\;\mathrm{conversion}\;\mathrm{rate}\;(\mathrm{FCR})\:=\:\mathrm{feed}\;\mathrm{intake}\;(\mathrm g)/\mathrm{weight}\;\mathrm{gain}\;(\mathrm g)$$

$$\mathrm{Weight}\;\mathrm{gain}\;(\mathrm g)\:=\:\mathrm{Average}\;\mathrm{final}\;\mathrm{body}\;\mathrm{weight}\:-\:\mathrm{Average}\;\mathrm{of}\;\mathrm{initial}\;\mathrm{weight}$$

$$\mathrm{Weight\ gain\ rate}(\%)\:=\:(\mathrm{Average\ body\ weight}-\mathrm{Average\ initial\ body\ weight})\backslash\mathrm{Average\ of\ initial\ body\ weight}.$$

$$\mathrm{The}\;\mathrm{specific}\;\mathrm{growth}\;\mathrm{rate}\;(\mathrm{SGR})\:=\:(\mathrm{Ln}\;\mathrm{Final}\;\mathrm{weight}\:-\:\mathrm{Ln}\;\mathrm{Initial}\;\mathrm{weight})/\;(\mathrm{No}\;\mathrm{of}\;\mathrm{days}\;\mathrm{in}\;\mathrm{trial})\:\times\:100$$

$$\mathrm{Survival}\;\mathrm{rate}\;(\mathrm{SR})\:=\:\mathrm{No}.\;\mathrm{of}\;\mathrm{life}\;\mathrm{fish}/\mathrm{Total}\;\mathrm{No}.\;\mathrm{of}\;\mathrm{fish}\:\times\:100$$

### Sexing of fish

Each treatment group, including the control, was rendered comatose after 30 days post feeding with phenoxy-ethanol (1:20,000, v/v) (Ambion, USA) [20], and then all fries were killed. Microscopic evaluations of gonad tissue were carried out using the standard acetocarmine squash method to determine the sex ratio following each treatment, [21].

### Estimation of sex hormones

The acquired samples of the fry were placed in a phosphate buffer solution with a saline pH of 7.4. The levels of many hormonal indices, including total testosterone, FSH, LH, and estrogen, were determined using an enzyme-linked immunosorbent assay (ELISA kits; DRG instrument GmbH) according to [22].

### Estimation of sex related genes by real-time PCR

In order to promote male sex differentiation, four sex genes were expressed in Nile tilapia fry. The Table below [23] lists the primers that were utilized in this study. Nile tilapia fries head samples were given when they were 14 days old, then fixed in RNA Llater (Ambion, USA) and kept at -80 ^₀^ C for the *Amh* expression in fries’ brain. At the end of the experiment, the trunk portion from fries was dissected to separate the gonads. According to the company instructions, the gonads were then preserved in RNA Later (Ambion, USA) and kept at -80 ^₀^ C for the expression of *Foxl2, Dmrt1,* and *Sox9*. Quantitative real-time PCR was performed on specific genes extracted from the head and gonads of all treated groups as well as the control group. Using the RNeasy Mini Kit (Qiagen, USA) and the manufacturer's instructions, total RNA was extracted. Gene-specific primers were employed in qPCR to measure variations in the expression levels of the genes and listed in Table 1. Cycling conditions for SYBR green real time PCR according to Quantitect SYBR green PCR kit. The Trizol (Invitrogen, USA) technique (1 ml Trizol/50 mg sample) was used in this study. Following [24], extracted RNA was quantified using Nano-Drop spectrophotometry, the 260/280 nm ratio was evaluated, and purity was verified at 1.80:2.00. Reverse transcription of c DNA was performed with a commercial kit (Invitrogen, USA). The test run included one cycle at 94 °C for 15 min, 40 cycles at 94 °C for 5 min, 62 °C for 30 s, and 72 °C for 30 s. The software strata gene MX3005P was used to determine CT values and amplification curves. The CT of each sample was compared with that of the control group in order to quantify the variance of gene expression on the RNA of the various samples according to the "ΔΔCt” method stated by [25] using the following ratio: (2^−DDct^). Whereas ΔΔCt = ΔCt reference – ΔCt target.

**Table 1:** Quantitative real-time PCR was performed using primer sequences for four sex genes

Primers	Sequences	References
***Amh***	**F: CACCCAGCTGCAGTACACGTAT** **R: TCAAAGGTCAACGTGATTGTTCC**	**Poonlaphdecha et al.,** [26]
***Foxl2***	**F: AAG AGG AGC CGG TTC AGG ACA A** **R: GCT CTC CCG GAT AGC CAT GG**	**Ijiri et al.,** [13]
***Dmrt1***	**F: CGG CCC AGG TTG CTC TGA G** **R: CCA ACT TCA TTC TTG ACC ATC A**	**Ijiri et al.,** [13]
***Sox9***	**F: CGG GAG AGC ATT CAG GTC AGT C** **R: CAG CTT TGC TGG AGG GAA GG**	**Ijiri et al.,** [13]

$$\triangle Ct\;target\;=\;Ct\;control\;-\;Ct\;treatment\;and\;\triangle Ct\;reference\;=\;Ct.control-\;Ct.treatment$$

### Histology of gonads

Standard histology procedure was followed according to 55. Gonads were obtained from the experimental Nile tilapia and preserved in fixative (10% buffered formalin) then dehydrated in a sequence of increasing alcohol, cleaned, and embedded with paraffin wax. Hematoxylin and Eosin (H & E) was used to stain 5 μm slices, which were then histologically analysed using a digital imaging system (Leica, Germany).

### Statistical analysis

All data are expressed as means and standard error. Results that had % values for count data received an angular transformation for further examination. A one-way analysis of variance was used to look at treatment impacts on different parameters after a Q-Q plot was used to confirm normality (ANOVA). SPSS version 16 for Windows was used to conduct all statistical analyses. Statistical significance was set at *p* < 0.05.

## Results

### Determination of the parameters of growth

**Fig. 1:**
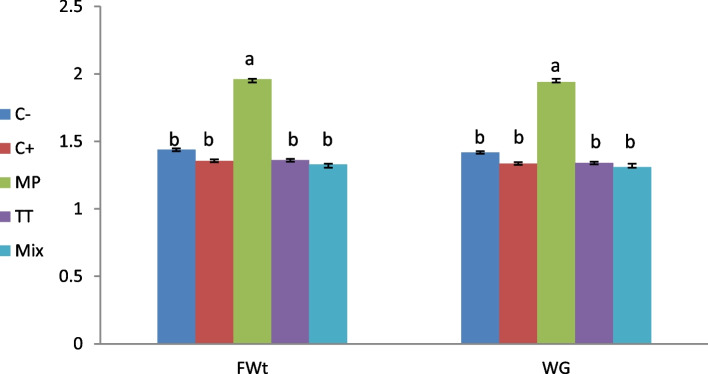
Final weight (FWt) and weight gain (WG) in *O. niloticus* after 30 days post feeding with different diet according to treated groups. Data are presented as mean ± SE (*n* = 9). The values with different superscript letters are significantly different (*P* < 0.05). C- is control negative group (not received any dietary supplements), C + is MT group received a diet containing 60 mg MT/kg feed, MP group received 2.0 g M*. pruriens*/kg feed, TT group received 2.0 g* T*. *terrestris*/kg feed, Mix group receive mixture of 1.0 g TT + 1.0 g MP/ kg feed

**Fig. 2:**
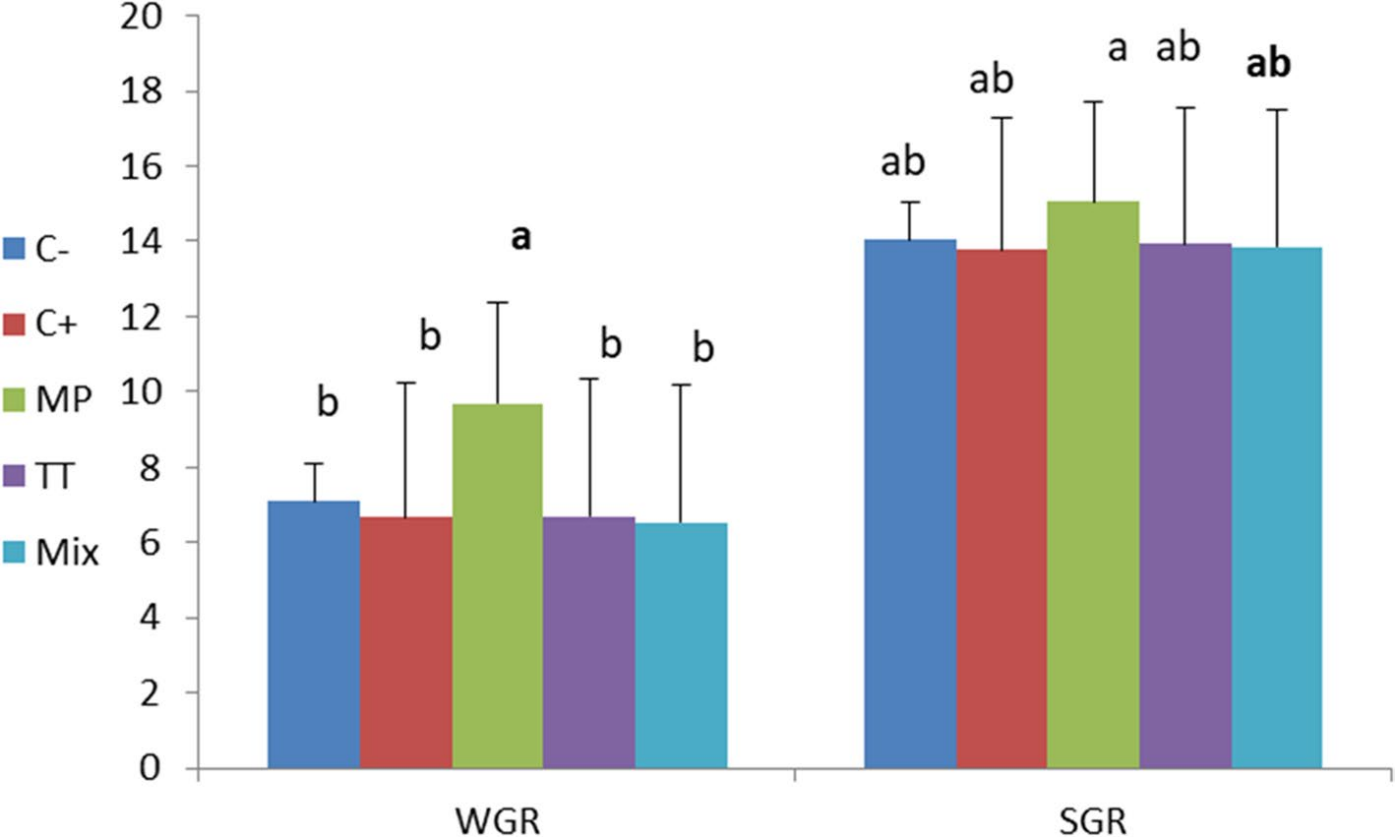
Weight gain rate (WGR) and specific growth rate (SGR) in *O. niloticus* after 30 days post feeding with different diet according to treated groups. Data are presented as mean ± SE (*n* = 9). The values with different superscript letters are significantly different (*P* < 0.05). C- is control negative group (not received any dietary supplements), C + is MT group received a diet containing 60 mg MT/kg feed, MP group received 2.0 g M*. pruriens*/kg feed, TT group received 2.0 g T*. terrestris*/kg feed, Mix group receive mixture of 1.0 g TT + 1.0 g MP/ kg feed

**Fig. 3:**
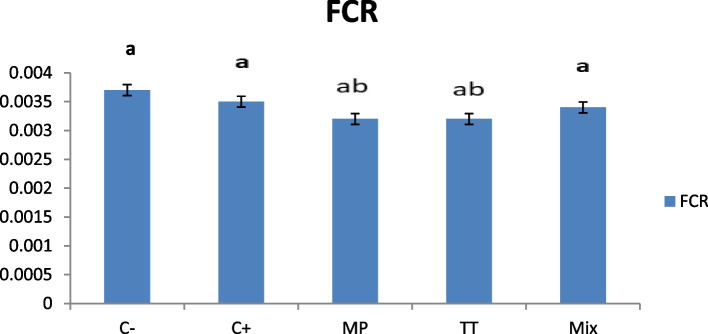
Feed convertion ratio (FCR) in *O. niloticus* after 30 days post feeding with different diet according to treated groups. Data are presented as mean ± SE (*n* = 9). The values with different superscript letters are significantly different (*P* < 0.05). C- is control negative group (not received any dietary supplements), C + is MT group received a diet containing 60 mg MT/kg feed, MP group received 2.0 g M*. pruriens*/kg feed, TT group received 2.0 g T*. terrestris*/kg feed, Mix group receive mixture of 1.0 g TT + 1.0 g MP/ kg feed

The effects of using herbal extract each alone or combination in compare with 17α methyl testosterone are displayed in Figs. 1 and 2. The growth parameters (final weight (FWT), weight gain (WG), weight gain rate (WGR), and specific growth rate (SGR)) were significantly (*p* < 0.05) improved in group treated with MP in comparison with all other dietary treatment groups and the control group. In addition, the best feed conversion ratio was recorded in MP group in comparison with all dietary treatment groups and the control group as presented in (Fig. 3).

### Determination of survival rate and sex ratio

As presented in Table 2, treatment with plant extract (TT or MP) has resulted in significantly higher (*P* < 0.05) survival percentage (88.5–89.5%, respectively) in comparison with the control, MT, and mixed treated group. Fish fed on diets containing plant extract (TT or MP) showed a significantly higher (*P* < 0.05) percentage of males (91.53–92.74%, respectively) compared to fish fed on the control diet (38.62%)and nearly similar to group treated with MT (93.21%).

**Table 2:** Estimation of the survival rate and sex ratio at the conclusion of the experiment (30 days post feeding)

Groups	Total no. of fries/group	No. of females	% of females	No. of males	% of males	Mortality rate %	Survival rate %	production
**C -**	200	89	61.38	56	38.62	27.5	72.5	208.51
**C + **	200	11	6.79	151	93.21	19	81	219.672
**MP**	200	13	7.26	166	92.74	10.5	89.5	350.84
**TT**	200	15	8.47	162	91.53	11.5	88.5	240.72
**Mix**	200	17	10.18	150	89.8	16.5	83.5	222.11

*C-* control negative group, *C* + control positive group MT (a diet containing 17α-methyl testosterone), *MP M. pruriens* group*,* *TT T. terrestris* group, *Mix* mixed group of *M. pruriens* and *T. terrestris*

### Determination of sex hormones

As presented in Table 3, treatment with plant extract (TT or MP) resulted in significantly higher (*P* < 0.05) total testosterone mainly after 30 days of feeding in comparison with the control and mixed group and nearly similar to MT group. Fish fed on diets containing plant extract (TT or MP) showed a significantly lower (*P* < 0.05) estrogen, LH and FSH compared to fish fed on the control diet and nearly similar to group treated with MT Determination of sex genes:

**Table 3:** Estimation of sex hormones in Nile tilapia fries gonads at 15 and 30 days post feeding

Groups	Period	Estrogen (pg/ml)	FSH (mIU/ml)	LH (mIU/ml)	Total Testosterone (mIU/ml)
**C –**	**(15 days)**	7.93 ^a^ ± 25.55	0.097 ^a^ ± 0.056	0.346 ^a^ ± 0.13	0.29 ^b^ ± 0.05
	**(30 days)**	9.82 ^a^ ± 75.2	0.38 ^a^ ± 0.02	1.44 ^a^ ± 0.086	1.07 ^bc^ ± 0.34
**C + **	**(15 days)**	3.89 ^bc^ ± 14.06	0.09 ^a^ ± 0.028	0.346 ^a^ ± 0.04	0.29 ^b^ ± 0.04
	**(30 days)**	5.25 ^b^ ± 63.9	0.193 ^b^ ± 0.03	1.34 ^ab^ ± 0.132	1.6 ^ab^ ± 0.2
**MP**	**(15 days)**	2.39 ^d^ ± 20.67	0.13 ^a^ ± 0.037	0.23 ^a^ ± 0.05	0.42 ^ab^ ± 0.086
	**(30 days)**	4.64 ^c^ ± 1.67	0.06 ^c^ ± 0.026	1.056 ^b^ ± 0.034	2.056 ^a^ ± 0.147
**TT**	**(15 days)**	3.25 cd ± 28.28	0.123 ^a^ ± 0.012	0.22 ^a^ ± 0.04	0.25 ^b^ ± 0.078
	**(30 days)**	5.13 ^b^ ± 46.8	0.086 ^c^ ± 0.039	1.04 ^b^ ± 0.017	1.38 ^ab^ ± 0.387
**Mix**	**(15 days)**	4.66 ^b^ ± 54.31	0.22 ^a^ ± 0.045	0.32 ^a^ ± 0.046	0.59 ^a^ ± 0.12
	**(30 days)**	6.21 ^b^ ± 53.95	0.14 ^bc^ ± 0.006	1.21 ^ab^ ± 0.115	0.78 ^c^ ± 0.015

Values are expressed as mean value (*n* = 30) ± SE

*C*- control negative group, *C* + control positive group MT (a diet containing 17α-methyl testosterone), *MP M. pruriens* group*,* *TT T. terrestris* group, *Mix* mixed group of *M. pruriens* and *T. terrestris.* *FSH* follicle stimulating hormone, *LH* luteinizing hormone

^a, b, c^Mean values with different superscript letters are significantly different (*P* < 0.05)

As presented in Table 4, the *Amh* gene 15 days post-feeding was expressed in the brain of Nile tilapia fries with higher levels in groups treated with MP, TT, and MT than the control and mixed groups. Additionally, as presented in Table 5, expression of the *Sox9* and *Dmrt1* genes in the gonads of fish fries revealed significantly higher levels in groups fed on MP, TT, and MT compared to control group after 30 days of feeding, whereas *Foxl2* gene expression was significantly lower in fish fed on MP, TT, and MT corporate diet compared to other groups.

**Table 4:** Determination of *Amh* gene in brain of Nile tilapia fries at 15 days post feeding

Groups	*Amh*
C -	3.89 ^c^ ± 13.94
C +	9.07 ^a^ ± 29.21
MP	8.85 ^a^ ± 56.52
TT	7.87 ^ab^ ± 54.61
Mix	6.95 ^b^ ± 29.27

Values are expressed as mean value (*n* = 30) ± SE

*C-* control negative group, *C* + control positive group MT (a diet containing 17α-methyl testosterone), *MP M. pruriens* group, *TT T. terrestris* group, *Mix* mixed group of *M. pruriens* and *T. terrestris*

^a, b, c^Mean values with different superscript letters are significantly different (*P* < 0.05)

**Table 5:** Estimation of sex genes expression in gonads of Nile tilapia fries at 30 days post feeding

Groups	*Foxl2*	*Sox9*	*Dmrt1*
C -	607.55 ^a^ ± 36.39	2.18 ^c^ ± 60.67	367.91 ^b^ ± 61.63
C +	264.84 ^b^ ± 54.67	5.82 ^a^ ± 69.94	679.47 ^a^ ± 38.63
MP	243.72 ^b^ ± 63.41	9.004^a^ ± 3.92	842. 006 ^a^ ± 36.008
TT	290.63 ^b^ ± 32.28	9.51 ^a^ ± 37.92	695. 04 ^a^ ± 35.62
Mix	376.22 ^b^ ± 44.26	6.34 ^b^ ± 65.69	439.65 ^b^ ± 68.57

Values are expressed as mean value (*n* = 30) ± SE

*C-* control negative group, *C* + control positive group MT (a diet containing 17α-methyl testosterone), *MP M. pruriens* group, *TT T. terrestris* group, *Mix* mixed group of *M. pruriens* and *T. terrestris*

^a, b, c^Mean values with different superscript letters are significantly different (*P* < 0.05)

### Histological analysis confirmed the masculinization of the gonads

**Fig. 4:**
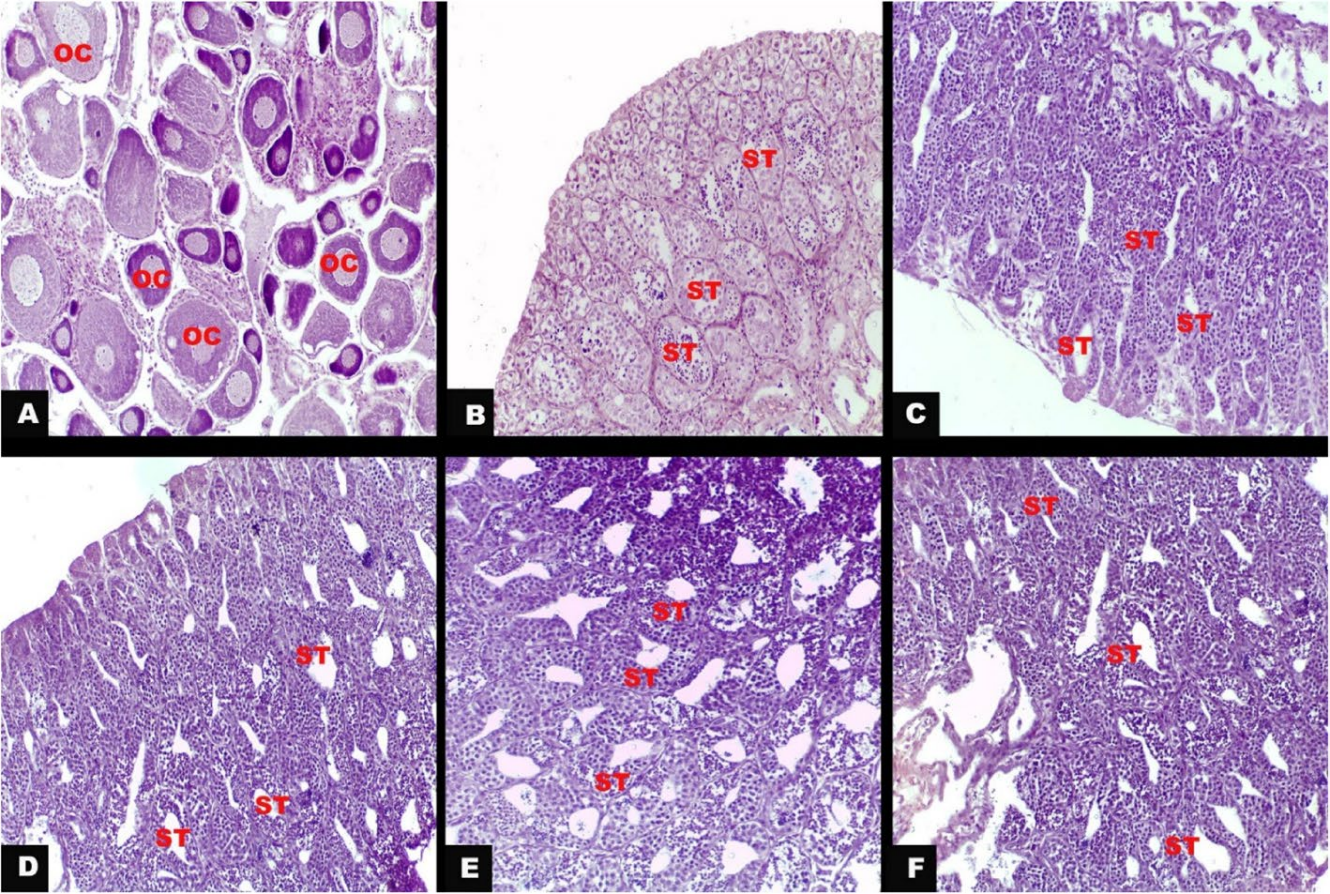
The histological features of Nile tilapia gonads at 30-day post hatching. **A** ovary of untreated fish (negative control group) showing numerous round oocytes (OC). **B** testis of untreated fish showing the normal structure of seminiferous tubules (ST). **C**, **D**, **E** & **F** testes obtained from MT, MP, TT, and the mixture group, respectively, showing the normal structure of seminiferous tubules (ST). (H &E; X200)

Histology of gonads was performed to identify sex after 30 days of treatment. Histological examination of the gonads of untreated fish (negative control group) exhibited typical female attributes that were confirmed by the presence of numerous round oocytes (Fig. 4A), as well as typical male characteristics that were confirmed by the presence of normal structure of seminiferous tubules (Fig. 4B). However, the fish treated with the compounds MT, MP, TT, and the mixture all exhibited solely male reproductive traits. Their gonads showed the normal structure of seminiferous tubules, without any female characteristics present (Fig. 4C, D, E and F). Overall, no noticeable damages or abnormalities were observed in the testicular and ovarian structures.

## Discussion

Nutraceuticals have been extensively used nowadays in aquaculture as growth promotors, immunity enhancers and production improvements. They are selected for their safety and efficiency than chemotherapeutics [27]. Based on the current findings; addition of each of the extracts of *T. terrestris* or *M. pruriens* seeds extract to feeds for Nile tilapia fry boosts mono-sex production, growth performance and survival rate in compare with MT treatment. We observed that *T. terrestris* and *M. pruriens* extract treatments at lower dosages (2 g/kg feed) had the potential to be employed as a supplement in fish diets, in particular the MP which was superior to TT in enhancement of growth performance. These results may be comparable to those previously observed [28], who found that nutritional treatment with *M. pruriens* extract at a concentration of 2.0 g/kg feed for 30 days may be used to produce a population of tilapia that is virtually entirely male with increased growth performance indicators. Therefore, sustainable, economical, and secure monosex fish production can be achieved by using medicinal plants in fish [24]. The increased growth performance observed in our study particularly in MP-treated group could be attributed to the palatability and digestibility of nutrients like proteins and carbohydrates found in plant extract that reflected on improvement of growth rate [29]. The results also demonstrated that treatment with the plant powder TT did not induce improvement in growth performance indicators compared to MP-treated group. This could be attributed to the antinutritional factors (hydrocyanic acid, phytate, nitrate, and oxalate) identified in TT leaves [30, 31]. 

The significant higher rates of survival caused by each of the plant extract than MT was not surprising These results were previously mentioned in different other studies [32–34]. According to the findings, the plant extract may not have had a negative impact on fish health, and therefore promoting the fish survival rate*.* Similarly, 100% survival rates were seen in fish fed meals containing 1 and 2 g/kg of powdered TT [7]. On the other hand, [35] observed that sex reversal with MT hormone caused 20% mortalities while sex reversal in fries was less than 80%. This increased survival rate by the plant extract could be attributed to the therapeutic properties related to its content of different vitamins such as vitamins A, C, and E, fatty acids, and essential amino acid [36].

In comparison to the mixed and the control groups, the dietary supplementation with each of the plant extract at the dose of 2.0 g/kg feed had the highest proportion of males (97.43± 0.13). In *TT* ethanol extract, the androgenic bioactive phytoconstituent protodioscin, a steroidal saponin, may be present and responsible for the increase of male sex ration [37]. Nevertheless, the specific process via which the plant extract creates the masculinization process and sex reversal is yet unknown, [38, 39]. Two pathways were postulated [40]. The first one proposed that phenols and steroidal saponins content in the plants, could be involved in activation of the endocrine system of fish, therefore stimulating the sexual differentiation and promote fish growth and survival [41]. The second proposed that the aromatase inhibitory activity of the plant may be a suitable source for the induction of masculinization [42, 43].

Hormonal profile showed a dramatic increase in total testosterone levels with decreasing the estrogen levels in tilapia groups treated with either MP or TT. These findings coincided with those previously reported [24]. It has been found that Tribulus contains Phenol, 2,4-bis (1,1-dimethylethyl), a potent aromatase inhibitor at both mRNA and protein level which considered as the major bioactive component with androgenic potential [20].

Different studies [23, 44, 45] asserted the difference in the expression of the reference genes between males and females, which may be explained by the cessation of expression that takes place during spermiogenesis as a result of chromatin formation or transcription factors that prevent the transcription of specific genes. Therefore, a difference in the expression of the reference genes may account for the statistically significant difference in the expression of the *Amh, Foxl2, Dmrt1*, and *Sox9* genes between males and females. In our study, the expression of the *Amh* gene in the Nile tilapia fry brain 15 days post-feeding was higher in MP, TT, and MT groups in comparison to the control and mixed groups. Males and females displayed different amounts of *Amh* expression at 15 days, with larger levels and sharper rises in the XY gonads [23]. The *Amh* (also known as Mullerian-inhibiting substance (Mis) or Mullerian inhibiting factor (Mif))) expression in the gonads of both sexes is responsible for repressing the development of müllerian ducts during testis differentiation and therefore promoting male sexing [46]. Therefore, the increased male sex ratio in our study could be attributed to the elevation of *amh* gene expression. In line with our results, a recent study proved that knocking out the *amh* gene by antisense RNA technology inhibited the testis formation in male sex tilapia [35].

The expression of *Sox9* and *Dmrt1* genes in gonads of fish fries 30 days post feeding was significantly increased whereas *Foxl2* gene expression was significantly decreased in MP, TT and MT in compare with control and mixed group The *Dmrt1* gene, a candidate for double expression in males, which is normally expressed at higher levels in males compared to females, may be responsible for these results. A more recent study showed that dmrt1 is the only male pathway gene tested indispensable for sex determination and functional testis development in tilapia [47] However, there are developmental stages of sturgeons in which the *Dmrt1* gene is not expressed at different levels, [23, 48, 49]. The *Foxl2* gene, on the other hand, is implicated in the start of female gonad development, [23]. Additionally, the fact that *Sox9* is expressed more strongly in the male gonads may mean that the gene is essential for the development of the testicles, [48]. In our study, the MP and TT powder enhanced the gene expression of male sex genes and inhibited the expression of female sex gene in comparable levels with MT. This result confirmed that plant extract can modulate the gene expression and thereby induce all-male sex in tilapia. the mechanism by which MP and TT increases the gene expression of male-related genes is yet elucidated since this is one the few reports discuss process on genetic level. However, the mRNA of the aromatase inhibitor, TT Phenol, 2,4-bis(1,1-dimethylethyl, present in some plant extract, such as TT is considered a strong bioactive mediator that stimulate the androgenic expressions [20].

Consistent with hormonal and gene expression profile, the histology confirmed that MT, MP, TT, and the mixture produced merely male reproductive traits with characteristic structure of seminiferous tubules without any female features. In addition no abnormalities or damage were detected.

Our results demonstrated a lesser efficacy of mixture of T.T. and M.P. than each of the T.T. or M.P. alone, particularly in growth performance of tilapia fries. The mechanism by which both herbal extracts are working either synergistic or antagonistic need more studies. However, previous studies on combination of herbal mixture including both MP and TT showed biochemical changes in kidney and liver of albino rabbit without any mortalities [50]. Moreover, the justification of doses used for dietary supplementation may be an important factor to have the desired effect in polyherbal formulation. In this regard, previous studies recommended that higher doses of polyherbal formulation should be avoided, and practitioners should cautiously prescribe because of the possible adverse effect [51]. Consistent with that, it has been postulated that dietary intervention with 2 g/Kg feed MP demonstrated the best growth performance than higher doses (4 and 6 g MP) [52]. Other factors could explain to polyherbal mixture is the form of the supplement. The results demonstrated by some studies showed that TT in powder from produced lower growth rate than TT plant extract [53]. TT is known to have antinutrient factors that hinder the bioavailability of some nutrients [54] and therefore their incorporation with MP could reduce the growth promoting effect.

A detailed future study is required to investigate the effect of polyherbal combinations on the induction of sex reversal, growth performance and survival rates in Tilapia aquaculture.

## Conclusion

The findings of this study indicate that dietary supplementation with each of the *T. terrestris* or *M. pruriens* seed extract alone at a concentration of 2.0 g/kg feed after 30 days feeding may be utilized to establish a population of exclusively male tilapia, improve growth performance and survival rate compared to combination between TT and MP and hormonal treatment with the synthetic steroid MT. This study paved the way of using the plant extracts as an alternative, safe and efficient method than the hormonal-induced monosex in Nile tilapia for future aquaculture industry. However, further investigations on using polyherbal mixture as alternatives in tilapia production are required.

## Data Availability

No datasets were generated or analysed during the current study.
